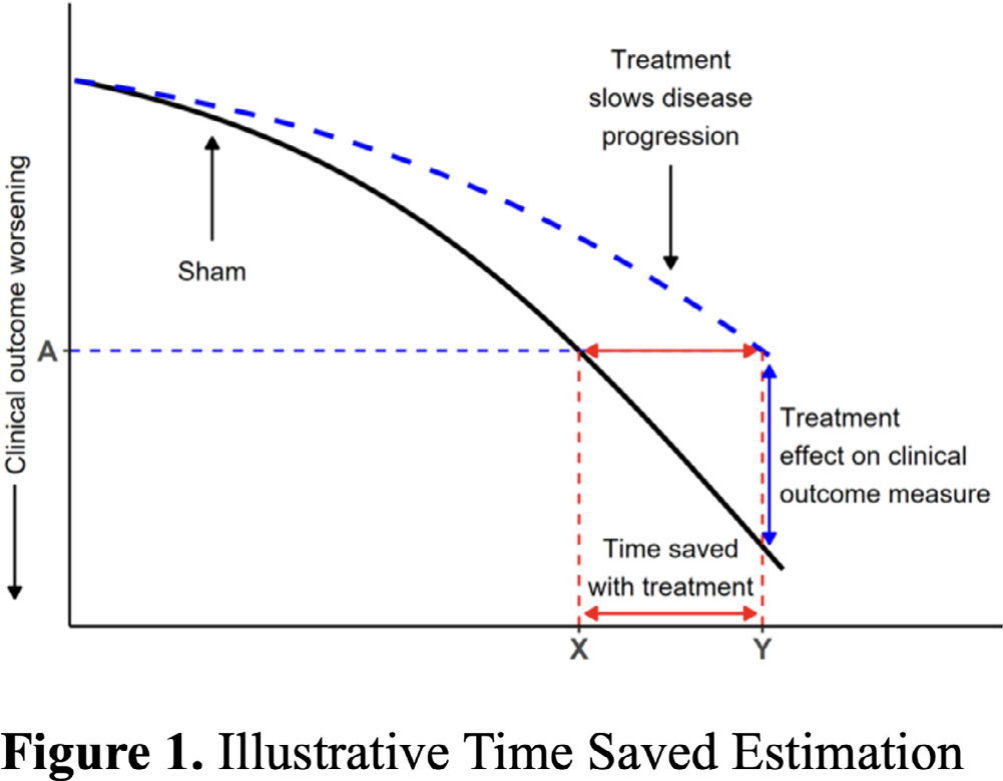# Spectris™ treatment is associated with significant time saved in the OVERTURE feasibility and open label extension (OLE) study

**DOI:** 10.1002/alz70861_108303

**Published:** 2025-12-23

**Authors:** Lily Lee, Ralph Kern, Marwan N. Sabbagh, Matthew Morgan, Joshua Christensen, Suzanne B. Hendrix

**Affiliations:** ^1^ Cognito Therapeutics, Cambridge, MA USA; ^2^ Banner Alzheimer's Institute, Phoenix, AZ USA; ^3^ Pentara, Salt Lake City, UT USA; ^4^ Pentara Corporation, Salt Lake City, UT USA

## Abstract

**Background:**

The OVERTURE (NCT03556280) randomized controlled trial (RCT)^1^ and open label extension (OLE) evaluated investigational evoked brain gamma oscillations (Spectris™), in mild‐moderate Alzheimer’s disease (AD). In the RCT, Spectris treatment was associated with significant reduction of decline in Alzheimer’s Disease Cooperative Study‐Activities of Daily Living (ADCS‐ADL), Mini‐Mental State Examination (MMSE) and Whole Brain Volume (WBV) versus sham treated participants. Time savings analysis allows for clinical interpretation of endpoints typically assessed in AD clinical trials^2^. We estimated time saved with Spectris treatment for ADCS‐ADL, MMSE and WBV in the OVERTURE RCT and OLE, representing up to 18 months of treatment.

**Method:**

OVERTURE compared one‐hour, daily, at‐home, treatment, active vs. sham (2:1 randomization) for six months. Participants had the option to enroll in the OLE for 12‐months of active treatment. Time saved in the active treatment group versus the sham group was estimated using mixed effects models. The estimated final value of the active arm for each outcome was projected horizontally onto the corresponding outcome’s sham progression (Figure 1). The time point at which the sham arm achieves that same value is subtracted from the final time point to create the “time saved” values.

**Result:**

In the RCT, 74 participants were randomized and 53 completed (33 active, 20 sham). A total of 44 participants entered the OLE and 30 completed. Time savings, measured in months, in ADCS‐ADL (4.83, *p* =0.0006), MMSE (4.56, *p* =0.1276) and WBV (4.09, *p* =0.0021) were observed in the RCT. Inclusion of the OLE resulted in time savings of 8.66, 9.93, 7.48 months over 14.64, 15.91 and 13.46 months of active treatment (all *p* < 0.0001) on ADCS‐ADL, MMSE, and WBV, respectively.

**Conclusion:**

The OVERTURE study demonstrated that Spectris treatment significantly reduced the decline of scale points in ADCS‐ADL, MMSE and WBV over 6 months compared to sham treatment. Evaluation of the OVERTURE RCT and OLE demonstrated significant time saved up to 18 months of treatment. These outcomes may be meaningful to Alzheimer’s patients and their caregivers and are being further evaluated in the ongoing HOPE pivotal trial of Spectris in mild‐moderate AD (NCT05637801).